# Flow diversion for unruptured fusiform aneurysms of the proximal middle cerebral artery

**DOI:** 10.3389/fneur.2023.1325983

**Published:** 2023-12-14

**Authors:** Yanting Gai, Maimaitiali Nuerdong, Yicheng Jiang, Wei Wang, Benfang Pu, Feng Xu, Donglei Song

**Affiliations:** ^1^Department of Neurosurgery, Shanghai Donglei Brain Hospital, Shanghai, China; ^2^Department of Neurosurgery, Huashan Hospital, Shanghai Medical College, Fudan University, Shanghai, China; ^3^National Center for Neurological Disorders, Shanghai, China; ^4^Shanghai Key Laboratory of Brain Function and Restoration and Neural Regeneration, Shanghai, China; ^5^Neurological Institute of Fudan University, Shanghai, China; ^6^Shanghai Clinical Medical Center of Neurosurgery, Shanghai, China

**Keywords:** flow diversion, aneurysm, fusiform, middle cerebral artery, endovascular treatment

## Abstract

**Background:**

Managing fusiform aneurysms of the proximal (M1) segment of the middle cerebral artery (MCA) is challenging due to difficulties in both surgical and endovascular treatment. In this study, we present our experience using flow diverter stents for managing unruptured M1 segment fusiform aneurysms.

**Methods:**

We conducted a retrospective review of the database of our institution to identify all patients who underwent flow diversion treatment for unruptured M1 segment fusiform aneurysms. We collected data on patient demographics, aneurysm characteristics, complications, angiographic follow-up results, and clinical outcomes.

**Results:**

A total of 10 patients (five male and five female patients) with 10 unruptured M1 segment fusiform aneurysms were included in the study. The average age of the patients was 48 years (range: 16–64 years); five patients had aneurysms smaller than 10 mm, four had aneurysms measuring between 10 and 25 mm, and one patient had an aneurysm larger than 25 mm. The successful deployment of flow-diverting stents was achieved in all cases. Procedure-related morbidity was observed in 10% of patients, but there were no deaths. All patients showed good outcomes (modified Rankin Scale score of 0–1); eight out of 10 patients had available follow-up angiography results with a mean follow-up period of 11.6 months (range: 6–24 months). Complete occlusion occurred in six out of eight reviewed cases (75%).

**Conclusion:**

Our preliminary findings suggest that using flow diversion for treating unruptured fusiform aneurysms in the proximal MCA is feasible and safe, with a satisfactory rate of complete occlusion. However, further studies involving larger case series are needed to validate the durability and efficacy of this treatment approach.

## Introduction

Fusiform intracranial aneurysms are rare and represent ~3–13% of all intracranial aneurysms. The most common affected site is the vertebrobasilar system, followed by the middle cerebral artery (MCA) (1). Fusiform MCA aneurysms typically occur more often in the proximal segment (M1) (2). These types of aneurysms are associated with challenges for surgical and endovascular treatment due to their connection with perforating lenticulostriate arteries. One useful technique for treating proximal MCA fusiform aneurysms is partial trapping combined with a high-flow bypass. However, there may be insufficient retrograde flow from the bypass to maintain these perforators open (1). A major issue with the conventional stents used in endovascular reconstruction is the recurrence of aneurysms. Flow diverters have recently shown promise in treating complex and fusiform MCA aneurysms (3–11), but few studies have specifically focused on those located in the M1 segment. In this study, we aimed to evaluate the safety and effectiveness of flow diversion for unruptured fusiform aneurysms located in the proximal MCA.

## Methods

### Patient selection

This retrospective study was approved by our institutional review board, and all patients provided general informed consent. We reviewed our database to identify unruptured fusiform aneurysms of the M1 segment of the MCA that were treated with flow diversion between July 2018 and May 2023. MCA aneurysms are typically classified into three types: proximal (M1 segment), bifurcation, and distal (12). The aneurysms were further categorized as saccular or fusiform based on their shape. Fusiform aneurysms were defined as those with an aneurysmal dilation exceeding 50% of the vessel wall circumference (13). We excluded unruptured fusiform M1 aneurysms associated with trauma, mycosis or infection, and inflammation from our study. Additionally, giant dolichoectatic or serpentine aneurysms were also excluded. Ten patients met these criteria and constituted the study population. We collected data on patient demographics, symptoms at presentation, postoperative angiograms, complications, clinical outcomes, and follow-up imaging.

### Antiplatelet agents

Patients received daily dual antiplatelet therapy consisting of 100 mg aspirin and 75 mg clopidogrel for 3 days prior to the procedure. Platelet function was assessed using thromboelastography (TEG) platelet mapping before proceeding with treatment. Patients who showed insufficient response to clopidogrel were switched to ticagrelor (180 mg) instead. Dual antiplatelet therapy was continued for 3 months after the procedure; thereafter, patients transitioned to aspirin monotherapy for a minimum of 6 months.

### Endovascular treatment

All procedures were performed under general anesthesia. Systemic heparinization was administered following sheath placement in order to prevent blood clotting during the procedure itself. A 6F shuttle (Cook) or an 8F Envoy guiding catheter (Cordis) was inserted into the relevant common carotid artery. Once a 5F Navien Intracranial Support Catheter (Medtronic Inc.) had been navigated to the distal internal carotid artery, a Marksman or Fastrack microcatheter was advanced over a 0.014-inch guidewire into the M2 segment of the MCA in preparation for flow diverter delivery. Two available flow diverters were used: Pipeline Embolization Device (PED) (Covidien, Irvine, California) and Tubridge (MicroPort Medical Company, Shanghai, China). The appropriate size and length of the flow diverter were selected based on the diameter of the parent artery and the size of the aneurysm. Under fluoroscopic guidance, the flow diverter was fully deployed. VasoCT was used to assess the correct apposition of the flow diverter to the vessel wall. For patients treated with adjunctive coil embolization, an Echelon-10 microcatheter (Medtronic Inc.) was advanced into the aneurysm via contralateral femoral artery access for coiling after implantation of the flow diverter.

### Evaluations and follow-up

Clinical evaluation took place immediately after each procedure as well as at discharge and during follow-up visits. Clinical outcomes were assessed using the modified Rankin Scale (mRS). Morbidity and mortality were defined as any deterioration greater than zero on mRS or any death related to treatment. CT angiography was performed 3 months post-procedure. Follow-up angiographies occurred initially at 6 months and then again at 12 months, followed by annual assessments until aneurysm occlusion occurred. Aneurysm occlusion during follow-up evaluations was evaluated according to the O'Kelly–Marotta Scale for Flow Diversion based on degree of filling: A—total filling; B—subtotal filling; C—entry remnant; D—no filling (14). The angiographic outcome of the jailed side branch was described as (1) patent (unchanged); (2) narrowing of diameter (stenosed); and (3) occluded.

## Results

### Population

This study included a total of 10 patients with unruptured fusiform M1 aneurysms, consisting of five male patients and five female patients. The average age of the patients was 48 years (ranging from 16 to 64 years). Among them, four patients presented with headaches, two with dizziness, and four had their aneurysm discovered incidentally. Fifty percent of the aneurysms were located on the right side. In terms of size, five patients had aneurysms smaller than 10 mm, four had sizes ranging from 10 to 25 mm, and one patient had a size larger than 25 mm. Table 1 provides a summary of the results.

**Table 1:** Demographics, aneurysm characteristics, clinical and radiological follow-up.

**Case no**.	**Age (yrs), sex**	**Clinical presentation**	**AN size (mm)**	**Treatment**	**Size of FD**	**Complication**	**FU angiographic result**	**FU angiographic time (mos)**	**mRS score at FU**
1	48/F	Dizziness	6^*^5^*^3	PED	3.0^*^18	No	OKM D	6	0
2	28/F	Headache	10^*^10^*^9	PED+coil	3.0^*^20	No	OKM D	24	0
3	60/M	Incidental finding	11^*^10^*^8	PED+coil	2.5^*^18	No	OKM D	15	0
4	52/F	Headache	6^*^5^*^4	PED	3.5^*^25	No	OKM C	6	0
5	44/M	Headache	15^*^12^*^12	PED+coil	3.5^*^20	No	OKM D	6	0
6	16/M	Headache	26^*^12^*^11	PED+coil	3.0^*^35	Infarction	OKM D	12	1
7	53/M	Incidental finding	5^*^3^*^2	TB	3.0^*^20	No	OKM C	18	0
8	57/M	Dizziness	8^*^7^*^7	PED	3.75^*^25	No	OKM D	6	0
9	64/F	Incidental finding	9^*^9^*^6	PED	3.0^*^25	No	NA	NA	0
10	61/F	Incidental finding	18^*^13^*^8	Surpass Evolve+coil	3.25^*^20	No	NA	NA	0

AN, aneurysm; FD, flow diversion; FU, follow-up; mRS, modified Rankin Scale; NA, not available; PED, pipeline embolization device; TB, tubridge.

### Feasibility

**Figure 1:**
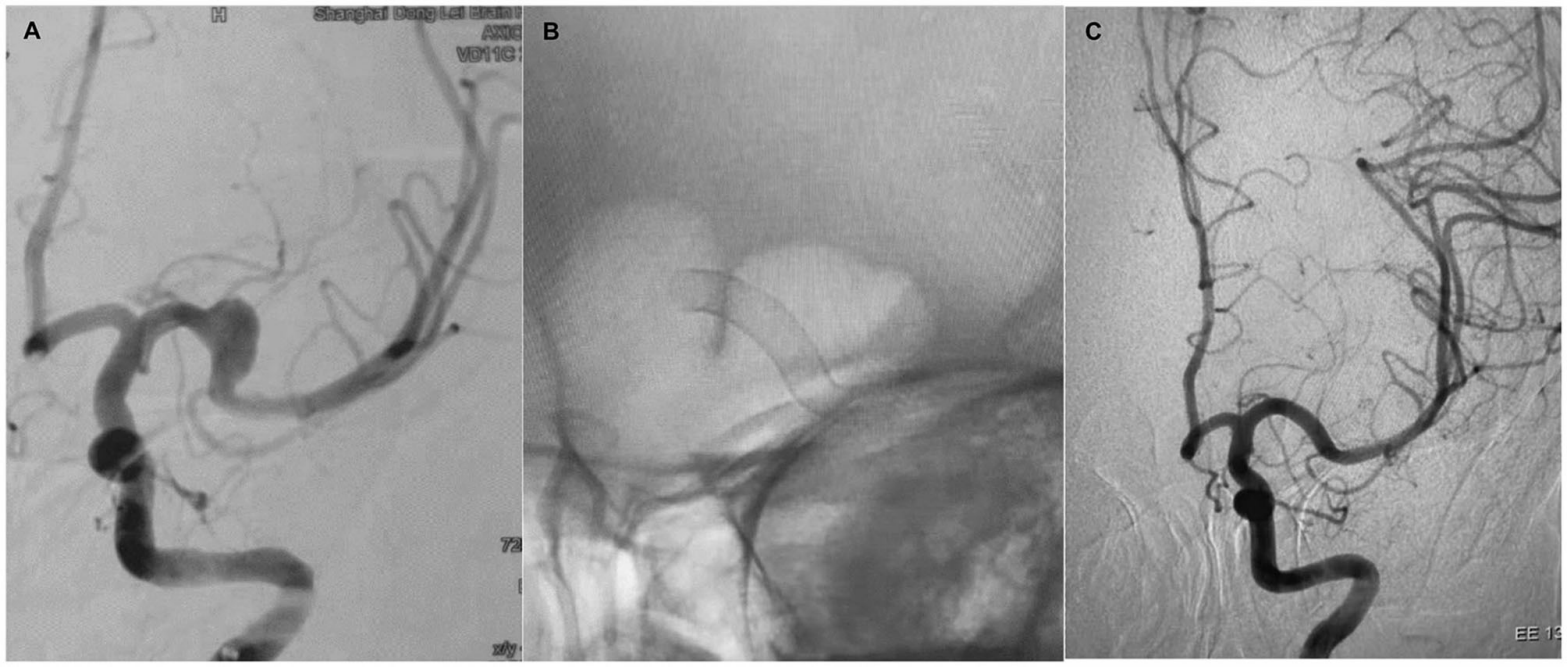
Case 1. A 48-year-old woman with a left-sided M1 fusiform aneurysm **(A)**. The patient was treated with a single PED **(B)**. Six-month follow-up showed complete occlusion of the aneurysm and remodeling of the vessel **(C)**.

**Figure 2:**
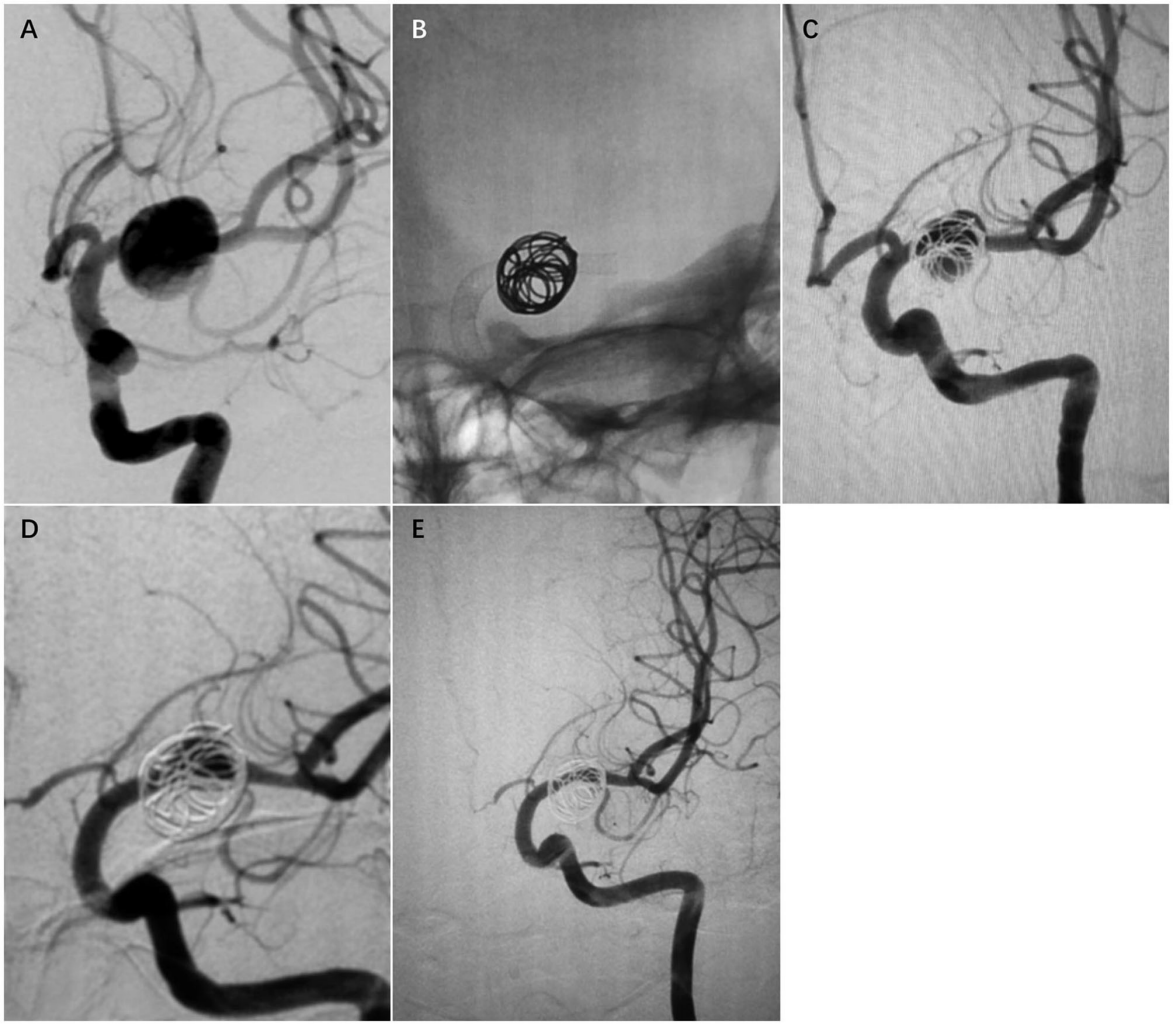
Case 2. A 28-year-old woman with a fusiform left MCA M1 segment aneurysm **(A)**. The patient was treated with a single PED and adjunctive coil embolization **(B)**. Immediate postoperative angiogram **(C)** showed stasis in the aneurysm sac. Angiograms obtained at the 6-month follow-up **(D)** and the 24-month follow-up **(E)** showed gradual total occlusion of the aneurysm with asymptomatic narrowing of the covered A1.

The delivery of the flow diverter was successful in all cases without any technical complications. A single flow diverter was used for each case: Eight aneurysms were treated with PED (80%), one with a Tubridge flow diverter (10%), and one with a Surpass Evolve flow diverter (10%) (Figure 1). Vessel wall apposition was achieved in all cases using the flow diverter. Adjunctive coil embolization was performed in five aneurysms due to their large size (Figure 2).

### Complications

**Figure 3:**
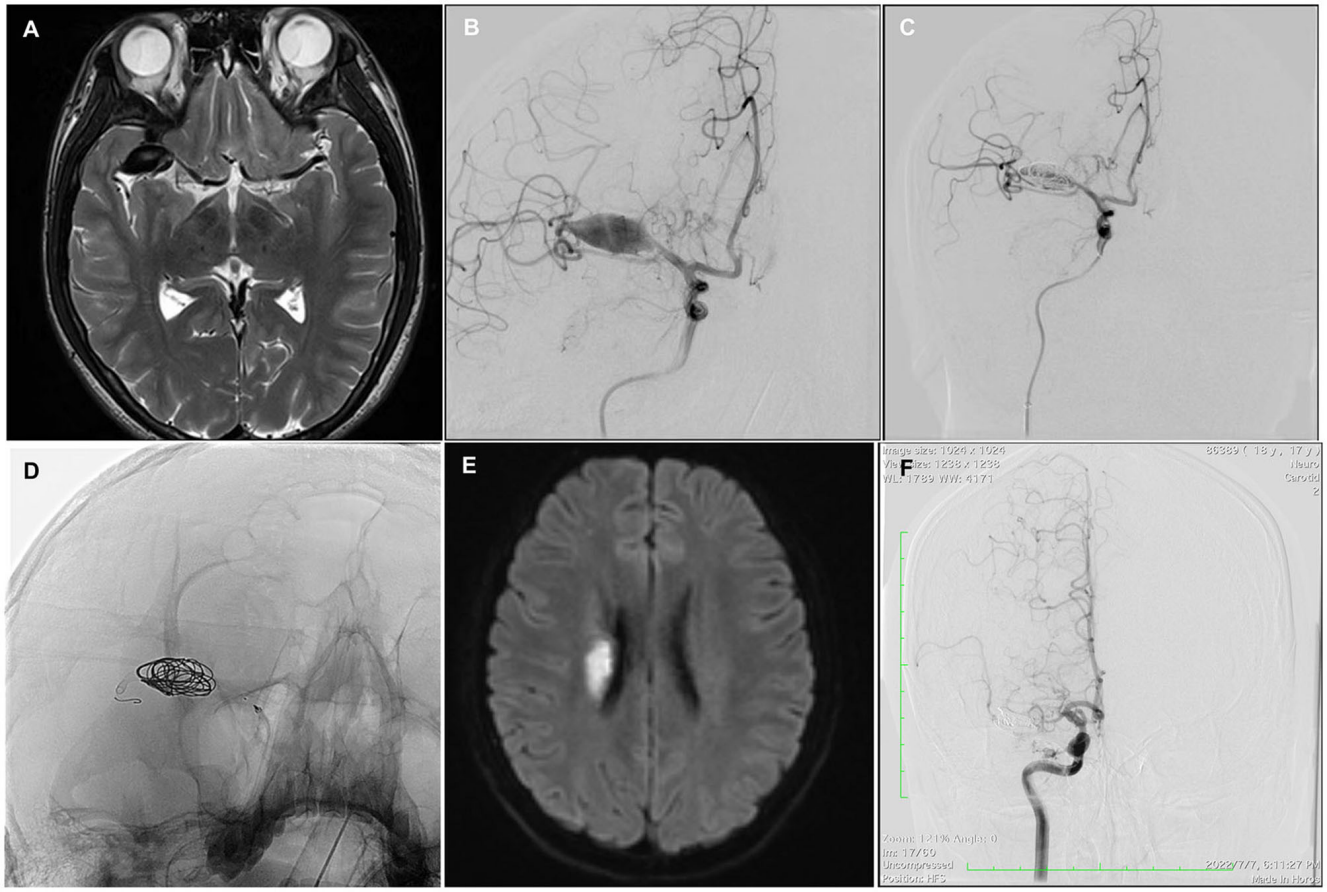
Case 6. A 16-year-old man with a giant fusiform right MCA M1 segment aneurysm, 26*12*11 mm in diameter **(A, B)**. The aneurysm was treated with a single PED and adjunctive coil embolization **(C, D)**; 6 h after the procedure, the patient developed an acute left hemiparesis. MR imaging showed acute infarction at the right corona radiata **(E)**. Twelve-month follow-up angiography showed a complete occlusion of the affected vessel **(F)**.

There were no intraprocedural complications. One patient (Case 6) awakened from the anesthesia without any neurological deficit; however, 6 h after the procedure, the patient developed an acute left hemiparesis. MR imaging showed acute infarction at the right corona radiata and MRA revealed patency of the device (Figure 3). The level of arachidonic acid percent inhibition decreased to a value of 20%. Immediate administration of low-molecular-weight heparin and a loading dose of cilostazol led to rapid improvement in this patient's condition. They were discharged with an mRS score of 1.

No hemorrhagic complications, aneurysm ruptures, or deaths occurred during the follow-up period. At clinical follow-up (mean 22.7 months; range 2-60 months), all patients (*n* = 10) showed good outcomes with mRS scores of 0–1. There were no procedural-related mortalities at the last follow-up.

### Angiographic follow-up

Eight out of ten patients had available angiographic data for review (mean 11.6 months; range 6–24 months). The remaining two patients have not yet undergone their first follow-up angiography. Overall, six out of eight aneurysms (75%) were completely occluded (OKM D), while two out of eight aneurysms (25%) had near complete occlusion (OKM C). Two (100%) A1 arteries covered by the flow diverter showed narrowing. One (25%) covered M2 branch exhibited asymptomatic occlusion, and one case presented with a reduced caliber. The patient who experienced acute infarction had complete occlusion in the affected vessel during follow-up. In one case, there was asymptomatic in-stent stenosis (<50%) in the initial portion of the flow diverter due to intimal hyperplasia, which did not progress according to the latest follow-up angiography results.

## Discussion

Fusiform aneurysms are a rare type of intracranial aneurysm that have an elongated shape. The unique shape of these aneurysms makes both surgical and endovascular treatments challenging. Standard microsurgical clipping or conventional coiling is not possible for fusiform MCA aneurysms due to the lack of a definable neck. Alternative treatments such as clip reconstruction, parent vessel occlusion with bypass, and endovascular reconstruction with conventional stents have been attempted, but their durability and efficacy are uncertain. It is also important to note that fusiform MCA aneurysms at the proximal (M1) segment are associated with lenticulostriate perforators.

Recently, flow diverters have emerged as a potential alternative for treating fusiform MCA aneurysms using endovascular methods (3, 5, 6, 8–11). Alturki et al. (10) described the successful treatment of two patients using the sequential coiling-assisted deployment of flow diverters, resulting in complete vessel reconstruction on follow-up angiography. Zanaty et al. (6), in their study involving 10 patients with complex MCA aneurysms treated with flow diverters, reported three cases where complete occlusion was achieved out of seven patients with fusiform aneurysms. Topcuoglu et al. (8), in another study, reported a high occlusion rate (75%) when using flow diversion for fusiform MCA aneurysms. In our series, we observed a final complete occlusion rate of 75% (6/8) during follow-up. Overall, these findings suggest that flow diversion may be a promising option for treating fusiform MCA aneurysms; however, further research is needed to determine its long-term effectiveness and safety.

Flow diverters are designed to influence the blood flow within an aneurysm by having a low porosity. However, the high metal coverage of these devices can reduce blood flow to the covered vessel and potentially lead to complications such as hemodynamic issues or thromboembolism. In a meta-analysis focused on flow diversion for MCA aneurysms, Cagnazzo et al. (15) reported a thromboembolic complication rate of 16.3%, with symptomatic stroke related to jailed branch occlusion and slow flow occurring in ~5% of cases. In another study by Lauzier et al. (16), which involved a multicenter cohort, pipeline embolization was used for proximal MCA aneurysms. Although there were no deaths or disabling strokes, there was still an 8% rate of non-disabling ischemic strokes observed. One concern when using flow diversion in the M1 segment is the potential occlusion of lenticulostriate perforators and subsequent significant ischemic events (17). However, in our series, none of our patients experienced clinical or radiographic evidence of symptomatic infarction due to the coverage of lenticulostriate arteries. One patient suffered acute infarction at the corona radiata due to aspirin resistance. Follow-up angiography showed that all lenticulostriate arteries and anterior choroidal arteries remained patent.

We believe that several important factors influence perforator patency during this procedure. First, selecting the appropriate size of the flow diverter is crucial; measuring the length and diameter of the affected artery using two-dimensional or three-dimensional DSA is mandatory (4). It is important not to undersize the stent as it may result in foreshortening and instead slightly oversize it for adequate positioning against the parent artery wall. Additionally, stretched pores and larger cells can help reduce perforator occlusion risk. Secondly, deploying the stent naturally in the M1 segment can help avoid dense packing of the device and reduce metal coverage (4). While ensuring adequate coverage of the aneurysm neck, it is important to avoid jailing the M2 branch (3). Finally, if adjunctive coil embolization is necessary for large or giant aneurysms, loose coiling is recommended. Loose adjunctive coiling recommended an intracavity embolic density of <12% (18). This approach can promote thrombus organization, accelerate aneurysm occlusion, and minimize interference with perforators. During follow-up in our study, two A1 arteries covered with flow diverters showed narrowing, and one M2 branch exhibited asymptomatic occlusion. These findings may be related to local hemodynamic changes and abundant collateral circulation. The blood flow from the contralateral anterior cerebral artery gradually becomes dominant when the anterior communicating artery opens up, while the posterior cerebral artery replaces blood supply to the inferior trunk of the MCA through cortical anastomosis branches.

One major concern regarding flow diversion for intracranial aneurysms is how to achieve complete aneurysm occlusion while reconstructing the parent artery and reducing postoperative complications. Adjunctive coil embolization has been suggested as a feasible option by some authors as it increases occlusion rates without raising periprocedural complication rates (19, 20). This approach can be particularly beneficial for large and giant intracranial aneurysms where there is rapid flow impact at the neck of the aneurysm; flow diverter-assisted coiling promotes occlusion while minimizing bleeding risks. The mechanisms behind this effect include inducing intra-aneurysmal thrombus formation due to the disturbance of coils within the aneurysm cavity, increasing the stability of flow diverters, promoting vascular endothelial proliferation, and achieving vascular remodeling (21). However, other studies have shown that adding coil embolization significantly prolongs procedural time and leads to higher neurological morbidity (22). In our study involving five large M1 fusiform aneurysms treated with adjunctive coil embolization, complete aneurysm occlusion was achieved in four patients according to follow-up angiography. The other one had undergone 3D-TOF MRA (23), but she is still waiting for angiography.

## Conclusion

Despite the limited number of cases in this retrospective study, our initial findings indicate that flow diversion is a feasible and safe treatment option for unruptured fusiform aneurysms of the proximal MCA. The complete occlusion rate was satisfactory. However, further research with a larger sample size is needed to validate the long-term effectiveness and durability of this treatment.

## Data availability statement

The original contributions presented in the study are included in the article/supplementary material, further inquiries can be directed to the corresponding authors.

## Ethics statement

The studies involving humans were approved by Shanghai Brain Donglei Group Hospital Review Board. The studies were conducted in accordance with the local legislation and institutional requirements. The participants provided their written informed consent to participate in this study. Written informed consent was obtained from the individual(s), and minor(s)' legal guardian/next of kin, for the publication of any potentially identifiable images or data included in this article.

## Author contributions

YG: Data curation, Investigation, Methodology, Resources, Formal analysis, Project administration, Writing—original draft. MN: Data curation, Investigation, Methodology, Writing—review & editing. YJ: Data curation, Investigation, Methodology, Writing—review & editing. WW: Data curation, Investigation, Methodology, Writing—review & editing. BP: Data curation, Investigation, Methodology, Writing—review & editing. FX: Conceptualization, Writing—original draft, Writing—review & editing. DS: Conceptualization, Supervision, Writing—review & editing, Validation.

## References

[1] Xu F Xu B Huang L Xiong J Gu Y Lawton MT. Surgical treatment of large or giant fusiform middle cerebral artery aneurysms: a case series. World Neurosurg. (2018) 115:e252–62. 10.1016/j.wneu.2018.04.031 29660547

[2] Seo D Lee SU Oh CW Kwon OK Ban SP Kim T. Characteristics and clinical course of fusiform middle cerebral artery aneurysms according to location, size, and configuration. J Korean Neurosurg Soc. (2019) 62:649–60. 10.3340/jkns.2019.0132 31591999 PMC6835147

[3] Burrows AM Zipfel G Lanzino G. Treatment of a pediatric recurrent fusiform middle cerebral artery (MCA) aneurysm with a flow diverter. J Neurointerv Surg. (2013) 5:e47. 10.1136/neurintsurg-2012-010478.rep 23188788

[4] Fischer S Perez MA Kurre W Albes G Bäzner H Henkes H. Pipeline embolization device for the treatment of intra- and extracranial fusiform and dissecting aneurysms: initial experience and long-term follow-up. Neurosurgery. (2014) 75:364–74. 10.1227/NEU.0000000000000431 24871140

[5] Monteith SJ Tsimpas A Dumont AS Tjoumakaris S Gonzalez LF Rosenwasser RH. Endovascular treatment of fusiform cerebral aneurysms with the Pipeline Embolization Device. J Neurosurg. (2014) 120:945–54. 10.3171/2013.12.JNS13945 24460489

[6] Zanaty M Chalouhi N Tjoumakaris SI Gonzalez LF Rosenwasser R Jabbour P. Flow diversion for complex middle cerebral artery aneurysms. Neuroradiology. (2014) 56:381–7. 10.1007/s00234-014-1339-x 24535072

[7] Ikeda DS Marlin ES Shaw A Powers CJ. Successful endovascular reconstruction of a recurrent giant middle cerebral artery aneurysm with multiple telescoping flow diverters in a pediatric patient. Pediatr Neurosug. (2015) 50:88–93. 10.1159/000375167 25790956

[8] Topcuoglu OM Akgul E Daglioglu E Topcuoglu ED Peker A Akmangit I. Flow diversion in middle cerebral artery aneurysms: is it really an all-purpose treatment? World Neurosurg. (2016) 87:317–27. 10.1016/j.wneu.2015.11.073 26723288

[9] Agnoletto GJ Aguilar-Salinas P Santos R Sauvagear E Hanel RA PED. Flex with Shield Technology: a feasible alternative for fusiform MCA aneurysms. Stroke Vasc Neurol. (2018) 3:185–8. 10.1136/svn-2017-000132 30294475 PMC6169605

[10] Alturki AY Schmalz PGR Ogilvy CS Thomas AJ. Sequential coiling-assisted deployment of flow diverter for treatment of fusiform middle cerebral artery aneurysms. Oper Neurosurg. (2018) 15:E13–8. 10.1093/ons/opx226 29140523

[11] Cimflova P Özlük E Korkmazer B Ahmadov R Akpek E Kizilkilic O. Long-term safety and efficacy of distal aneurysms treatment with flow diversion in the M2 segment of the middle cerebral artery and beyond. J Neurointerv Surg. (2021) 13:631–6. 10.1136/neurintsurg-2020-016790 33082291

[12] Zaidat OO Castonguay AC Teleb MS Asif K Gheith A Southwood C. Middle cerebral artery aneurysm endovascular and surgical therapies comprehensive literature review and local experience. Neurosurg Clin N Am. (2014) 25:455–69. 10.1016/j.nec.2014.04.005 24994084

[13] Sacho RH Saliou G Kostynskyy A Menezes R Tymianski M Krings T. Natural history and outcome after treatment of unruptured intradural fusiform aneurysms. Stroke. (2014) 45:3251–6. 10.1161/STROKEAHA.114.006292 25205312

[14] O'kelly CJ Krings T Fiorella D Marotta TR. A novel grading scale for the angiographic assessment of intracranial aneurysms treated using flow diverting stents. Interv Neuroradiol. (2010) 16:133–7. 10.1177/159101991001600204 20642887 PMC3277972

[15] Cagnazzo F Mantilla D Lefevre PH Dargazanli C Gascou G Costalat V. Treatment of middle cerebral artery aneurysms with flow-diverter stents: a systematic review and meta-analysis. Am J Neuroradiol. (2017) 38:2289–94. 10.3174/ajnr.A5388 28982785 PMC7963743

[16] Lauzier DC Root BK Kayan Y Almandoz JED Osbun JW Chatterjee AR. Pipeline embolization of proximal middle cerebral artery aneurysm: a multicenter cohort study. Interv Neuroradiol. (2022) 28:50–7. 10.1177/15910199211015578 33951971 PMC8905083

[17] Bhogal P Martinez R Gansladt O Bäzner H Henkes H. Management of unruptured saccular aneurysms of the M1 segment with flow diversion: a single center experience. Clin Neuroradiol. (2018) 28:209–16. 10.1007/s00062-016-0553-9 27942770

[18] Wang Z Tian Z Li W Wang J Zhu W Zhang M. Variation of mass effect after using a flow diverter with adjunctive coil embolization for symptomatic unruptured large and giant intracranial aneurysms. Front Neurol. (2019) 10:1191. 10.3389/fneur.2019.01191 31798519 PMC6874129

[19] Lin N Brouillard AM Krishna C Mokin M Natarajan SK Sonig A. Use of coils in conjunction with the Pipeline embolization device for the treatment of intracranial aneurysms. Neurosurgery. (2015) 76:142–9. 10.1227/NEU.0000000000000579 25255261

[20] Zhang Q Shao Q Chang K Zhang H He Y Andrade-Barazarte H. Safety and efficacy of coils in conjunction with the Pipeline Flex Embolization Device for the treatment of cerebral aneurysms. Front Neurol. (2021) 12:651465. 10.3389/fneur.2021.651465 34759878 PMC8573379

[21] Ravindran K Casebella AM Cebral J Brinjikji W Kallmes DF Kadivel R. Mechanism of action and biology of flow diverters in the treatment of intracranial aneurysms. Neurosurgery. (2020) 86:s13–9. 10.1093/neuros/nyz324 31838528 PMC6911734

[22] Park MS Kilburg C Taussky P Albuquerque FC Kallmes DF Levy EI. Pipeline Embolization Device with or without adjunctive coil embolization: analysis of complications from the IntrePED registry. Am J Neuroradiol. (2016) 37:1127–31. 10.3174/ajnr.A4678 26767709 PMC7963540

[23] Shao Q Li Q Wu Q Li T Li L Chang K. Application of 3D T1-SPACE combined with 3D-TOF sequence for follow-up evaluation of stent-assisted coil embolization for intracranial aneurysm. J Inter Med. (2021) 4:71–6. 10.1016/j.jimed.2021.02.007 34805951 PMC8562288

